# Impact of Additional Transthoracic Electrical Cardioversion on Cardiac Function and Atrial Fibrillation Recurrence in Patients with Persistent Atrial Fibrillation Who Underwent Radiofrequency Catheter Ablation

**DOI:** 10.1155/2016/4139596

**Published:** 2016-02-28

**Authors:** Deguo Wang, Fengxiang Zhang, Ancai Wang

**Affiliations:** ^1^Department of Gerontology, Yijishan Hospital of Wannan Medical College, Wuhu 241001, China; ^2^Department of Cardiology, The First Affiliated Hospital of Nanjing Medical University, China

## Abstract

*Backgrounds and Objective*. During the procession of radiofrequency catheter ablation (RFCA) in persistent atrial fibrillation (AF), transthoracic electrical cardioversion (ECV) is required to terminate AF. The purpose of this study was to determine the impact of additional ECV on cardiac function and recurrence of AF.* Methods and Results*. Persistent AF patients received extensive encircling pulmonary vein isolation (PVI) and additional line ablation. Patients were divided into two groups based on whether they need transthoracic electrical cardioversion to terminate AF: electrical cardioversion (ECV group) and nonelectrical cardioversion (NECV group). Among 111 subjects, 35 patients were returned to sinus rhythm after ablation by ECV (ECV group) and 76 patients had AF termination after the ablation processions (NECV group). During the 12-month follow-ups, the recurrence ratio of patients was comparable in ECV group (15/35) and NECV group (34/76) (44.14% versus 44.74%, *P* = 0.853). Although left atrial diameters (LAD) decreased significantly in both groups, there were no significant differences in LAD and left ventricular cardiac function between ECV group and NECV group.* Conclusions*. This study revealed that ECV has no significant impact on the maintenance of SR and the recovery of cardiac function. Therefore, ECV could be applied safely to recover SR during the procedure of catheter ablation of persistent atrial fibrillation.

## 1. Introduction

Atrial fibrillation (AF) is the most supraventricular arrhythmias which involved 0.4–1% of people in the general population [1]. AF lead to a low quality of life and high risk of heart failure, stroke, mortality, and rehospitalization [2–4]. Drug therapy is less effective in maintaining sinus rhythm in 40% of all patients [5] with high adverse effects. Nowadays, left atrial catheter ablation is widely used to treat AF [6, 7]. Pulmonary vein isolation (PVI) and complex fractionated atrial electrograms (CFAE) ablation are two common strategies to eliminate triggers and arrhythmogenic substrate of AF [8, 9]. Moreover, additional linear ablation lines, for example, at the left atrial roof and mitral isthmus, may abolish more substrate. However, there are considerable amounts of people who need to receive transthoracic electrical cardioversion (ECV) to terminate persistent AF even after ablation. It is not clear whether ECV affect the recovering of cardiac function and reoccurrence of AF after radiofrequency catheter ablation (RFCA). Therefore, the purpose of this study was to determine the impact of additional ECV on cardiac function after RFCA.

## 2. Methods and Materials

Patients with symptomatic drug-resistant persistent AF who underwent catheter ablation at our hospitals were included in this study. Persistent AF is defined as AF which is sustained beyond seven days, or lasting less than seven days but necessitating pharmacologic or electrical cardioversion [10]. Transthoracic echocardiography (TTE) was performed 3 times (before and 6 and 12 months after ablation) to measure conventional parameters and LA function. Ethics approval of the present study was obtained from the local review committee, and all patients provided written informed consent.

Echocardiographic study was performed by an observer who was blinded to the study design using an IE33 ultrasound machine (PHILIP, USA) with a 2.5 MHz transducer. Echocardiograms were recorded and analyzed offline using a customized software package (EchoPAC Systems, PHILIP, USA).

Extensive encircling pulmonary vein isolation (PVI) was performed at the atrial interface of the PV-left atrium [11]. A 7.5-Fr irrigation catheter with a 3.5 mm distal electrode (ThermoCool, Biosense Webster, USA) was used for ablation. An electroanatomical mapping system (Carto*™*, Biosense Webster, Diamond Bar, CA, USA) was used to validate that linear lines were continuous. The endpoint of the extensive PVI was creation of extensive bidirectional conduction block from the atrium to the PVs. If AF was sustained after PVI, additional ablation consisting of linear ablation of the LA roof, superior vena cava isolation, and/or ablation of continuous fractionated atrial electrograms was performed. If AF did not terminate after that additional ablation, SR was restored by transthoracic electrical cardioversion (100–200 J). Patients who did not restore SR were excluded from this study. Patients were then divided into two groups on the basis of transthoracic electrical cardioversion: electrical cardioversion (ECV group) and none electrical cardioversion (NECV group).

After ablation, patients were followed up for 12 months. At each outpatient visit, a 12-lead electrocardiogram (ECG), 24 hours' Holter, and echocardiographic study were performed. ECG and Holter also were done any time the patients reported palpitations. If the ECG showed any episodes of AF or any other atrial tachyarrhythmias lasting >30 s during follow-up, recurrence of AF was diagnosed.

Continuous data are expressed as mean ± SD. Categorical data are expressed as absolute numbers or percentages. Comparisons between groups were performed using independent samples* t*-test, and *χ* ^2^ test as appropriate. Two-sided *P* < 0.05 was considered significant for all analyses.

## 3. Results

A total of 111 patients (89 men; age 56 ± 11 years) were included in this study. Among them, 35 patients were returned to sinus rhythm after ablation by ECV (ECV group) and 76 patients had AF termination after the ablation processions (NECV group). As shown in Table 1, the clinical characteristics of the patients in the ECV and NECV groups were comparable. During the 12-month follow-ups, the recurrence ratio of patients was comparable in ECV group (15/35) and NECV group (34/76) (44.14% versus 44.74%, *P* = 0.853).

**Figure 1:**
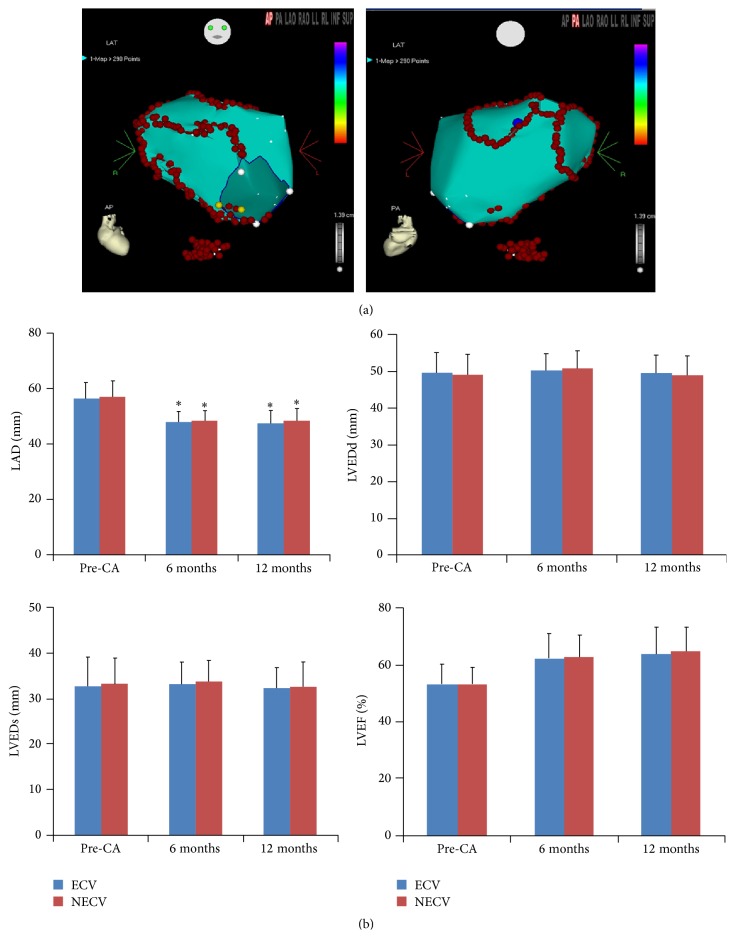
The three-dimensional diagram of catheter ablation persistent atrial fibrillation and myocardial biomarkers. Representative circumferential pulmonary vein isolation and additional ablation line on an electroanatomic map (a). Cardiac function by echocardiography (b). LAD: left atrial diameter. LVEDd: left ventricle diastolic end diameter. LVEDs: left ventricle systolic end diameter. LVEF: left ventricle ejection fraction. ^*∗*^ *P* < 0.01 versus pre-CA (before catheter ablation).

As shown in Figure 1(b), left atrial diameters (LAD) tent to decrease significantly compared with preablation in both ECV and NECV groups during the 6 and 12 months' follow-ups. There were no significant changes of LVEDs, LVEDd, and LVEF in both groups during follow-ups. Importantly, there were no significant differences in these parameters between ECV group and NECV group which reveal that ECV did retard the recovery of cardiac function (Figure 1(b)).

## 4. Discussions

This study had revealed that ECV during RFCA in patients with persistent AF did not affect recurrence of AF and LA and LV function in the long term follow-ups although LAD reduced significantly after ablation.

Recovering to SR was expected to achieve better outcome of persistent AF ablation [12]. However, it is controversial to use ECV to terminating AF [13, 14]. Faustino et al. [13] reported that termination of AF through atrial tachycardia during catheter ablation was more effective than both ECV and direct SR in maintaining stable SR. In contrast, Wang et al. [14] observed that long-term SR maintenance is not associated with the style of AF termination. Mont et al. [7] had revealed that repeatedly ECV could act as a predictor factor for ablation failure for long time. In this study, we found that the recurrence ratio was similar in ECV group (15/35) and NECV group (34/76) during the 12 months' follow-ups (Table 1). This finding suggested that the requirement of ECV to terminate AF was not a good indicator for high AF recurrence.

Different results had been reported about the changes of cardiac function after ablation. Previous study revealed that CA can reduce left atrial (LA) volume without a deleterious impact on contractile function [15]. In contrast, a recent study based on MRI imaging reported that LA contractility and compliance are markedly impaired years after successful AF ablation which is closely related to scar burden [16]. ECV causes a so-called phenomenon of “left atrial stunning” [17] which characterized that left atrial function does not recover and even decrease further in patients with AF or atrial flutter (AFL). Similar phenomena were reported in drug cardioversion and spontaneous termination of AF [18, 19]. In this study, LAD tent to reduction in both groups. Furthermore, ECV has no further and directed impact on cardiac function and LAD.

Taken together, our findings revealed that ECV has no significant impact on the maintenance of SR and the recovery of cardiac function. Therefore, ECV could be applied safely to recover SR during the procedure of catheter ablation of persistent atrial fibrillation.

## Figures and Tables

**Table 1:** Clinical characteristics and recurrence.

	ECV (35)	NECV (76)	*P* value
Demographics			
Age (years)	56 ± 12	56 ± 10	0.987
Male (%)	28 (80)	61 (77.6)	0.974
BMI (kg/m^2^)	26.3 ± 3.8	24.9 ± 2.7	0.647
Comorbidity, *n* (%)			
Hypertension (%)	12 (34.3)	23 (30.1)	0.672
Diabetes mellitus (%)	2 (5.7)	3 (3.9)	0.677
CHD (%)	2 (5.7)	4 (5.3)	0.922
Drugs			
ACE/ARB	6 (17.1)	13 (17.1)	0.996
*β*-blocker	13 (37.1)	29 (38.2)	0.509
AADs, class I	11 (31.4)	24 (31.6)	0.987
AADs, class III	23 (65.7)	50 (65.8)	0.993
Duration (years)	7.2 ± 6.1	5.5 ± 5.4	0.129
Recurrence (%)	15 (42.86)	34 (44.74)	0.853
